# G protein-mediated signal transduction: a molecular choreography of G protein activation after GTP binding

**DOI:** 10.1038/s41392-024-01903-3

**Published:** 2024-07-16

**Authors:** Evi Kostenis, Lars Jürgenliemke, Judith Alenfelder

**Affiliations:** 1 Department of Molecular-, Cellular-, and Pharmacobiology, Institute of Pharmaceutical Biology, University of Bonn, Bonn, Germany; 2 Graduate Training Group RTG2873, University of Bonn, Bonn, Germany

**Keywords:** Structural biology, Biological techniques

A recent report from the Skiniotis group,^1^ published in *Nature*, provides fascinating insight into the molecular choreography underlying a quintessential cellular signaling mechanism: G protein-mediated signal transduction. Using time-resolved cryo-EM, the authors pieced together many sequential structural snapshots to visualize how a trimeric αβγ G protein—after being convinced by its activated G protein-coupled receptor (GPCR) to release GDP—picks up GTP, to set in motion finely tuned conformational transitions that lead to the conversion into the active state and, finally, subunit dissociation as well as disengagement from the receptor.

Visualizing heterotrimeric G proteins in motion is a daunting challenge. Why face it? Because G proteins are the molecular machines that empower signal transduction of GPCRs, the largest family of transmembrane signaling proteins in the human genome.^2,3^ Located at the inner leaflet of the plasma membrane, G proteins “decode” the message encrypted in conformational changes of the intracellular receptor face that occur when receptors meet the fascinating variety of extracellular cues.

Animal cells express about 800 GPCRs but only four major G protein subclasses (Gi, Gs, Gq, G12). This apparent imbalance between signal detectors and signal transducers raises several fundamental questions. What are the molecular determinants governing specific or promiscuous G protein recognition? Where do primary determinants of GPCR:G protein coupling selectivity reside? How can G proteins of different subfamilies, that are so similar and conserved in structure, exert so many different signaling functions?

To answer those questions, knowledge about G protein activation mechanics is paramount, especially concerning the pivotal step in the regulated occurrence of G protein-mediated signaling: guanine nucleotide exchange, i.e., replacement of GDP for GTP on the G protein α subunit. In animal cells, the basal rate of nucleotide exchange is small; therefore, guanine nucleotide exchange factors (GEFs) such as GPCRs are needed to increase the reaction rates by several folds and to convert inactive GDP-bound Gα to active Gα-GTP. While great strides have been made in defining the structures of Gα subunits in GTP-bound, GDP-bound, and heterotrimeric states,^2,3^ viewing G proteins in their ON and OFF states only is not enough to understand how they function.

Activation of G proteins by nucleotide exchange is a highly dynamic, multistep process occurring on the millisecond timescale in living cells. GDP unbinding is the rate-limiting step of the Gα nucleotide cycle, which is followed immediately by GTP loading due to its 10-fold higher abundance in the cytoplasm. This makes the nucleotide-free G protein too short-lived an intermediate in the generation of the active signaling species, Gα-GTP, to visualize the conformational transitions in the living cell context.

Imagine being able to visualize—by structural biology techniques in real time—a G protein transitioning into its active state in a near-native environment, being able to relate structural transitions to G protein function, and even being able to capture highly relevant but short-lived conformational intermediates! The dream of visualizing the entire nucleotide-dependent switch as a 3D movie sounds like science fiction. Using cryo-EM with freeze trapping at different time points and technical tricks—detergent and decreased temperature—to slow down the process, a team lead by Georgios Skiniotis has now come close: They pieced together a sequence of molecular events visualizing precisely the part of this switch that underlies G protein activation in response to GTP binding.^1^

**Fig. 1:**
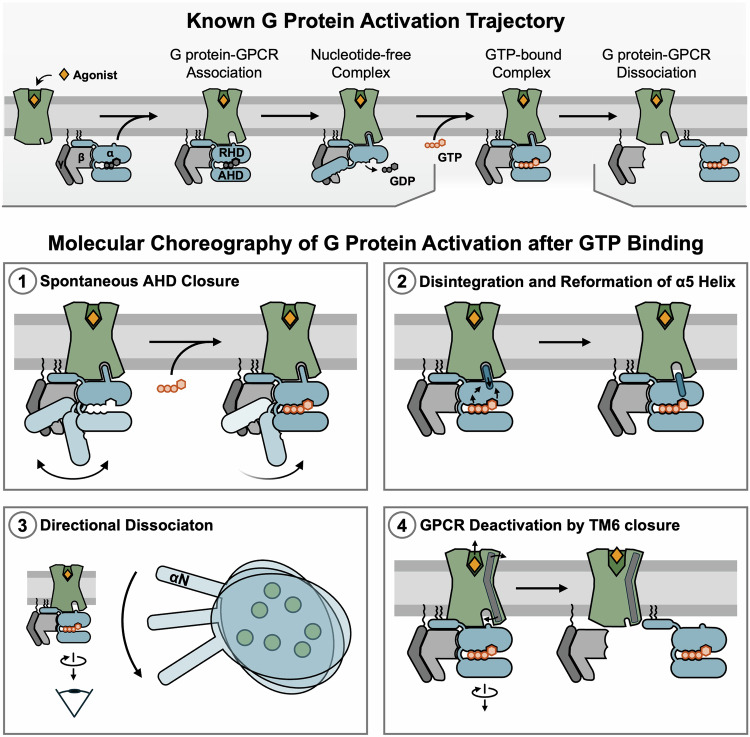
Four new aspects of the G protein activation choreography. Top. Known aspects of the G protein activation trajectory include ligand binding to the receptor and association of receptor and G protein, leading to the release of GDP from the Gα subunit. The high energy of GTP binding to Gα stabilizes an effector-complementary conformation that also prevents stable interaction with the Gβγ complex. Active Gα and Gβγ subunits dissociate and, finally, disengage from the receptor. (1) In the empty pocket state, the α-helical domain moves freely in between the open and closed conformations of Gα. Upon GTP binding, the AHD loses flexibility and becomes tightly apposed to the RHD, i.e., locked in the closed conformation. (2) As a result of the concerted conformational changes in various segments of Gα, the C-terminal α5 helix disintegrates and reforms with a shifted helical register, incompetent to maintain stable receptor contacts. (3) The loss of stable complementarity to the receptor contact surface results in the dissociation of the G protein in a directional manner with an anti-clockwise rotational movement when viewed from the cytosol. (4) Deactivation of the receptor (β2AR) follows instantly upon G protein activation by GTP binding. TM6 moves back inwards towards its position in the inactive state, while c-Epi, a conformationally constrained, highly efficacious β2AR agonist, relocates towards the extracellular entry of the ligand binding pocket

The team suggests a likely scenario for a multistep mechanism of Gs protein activation beginning with GTP binding to the open and empty Gαs, up to the disengagement of the G protein from the β2-adrenergic receptor (β2AR). Their results show nicely how GTP binding to the Ras-homology domain (RHD) of Gαs in an open, empty pocket conformation and subsequent closure of the α-helical domain (AHD) set in motion profound changes in the overall Gα conformation that finally result in G protein activation and its dissociation from the receptor. So far, we could only imagine what happens after GDP is kicked out of its binding pocket by an active GPCR and replaced with GTP. Now we have many structures at hand, reconstructing a G protein during activation, starting with the empty pocket, nucleotide-free state and following its conformational trajectory after GTP binding with unprecedented detail (Fig. 1). This molecular choreography contains several intriguing new features and provokes new thinking:

1. Opening and closure of the AHD in the empty pocket Gα happens stochastically. Upon binding of GTP, the closing movement remains stochastic. The re-opening, in contrast, is prevented, as the nucleotide bridges the interface between the AHD and the RHD to lock the α subunit in its closed conformation. Stochastic opening and closure are also observed in GDP-bound simulations, suggesting spontaneous and frequent rigid-body rotational movement of the AHD away from the RHD, even in the absence of an activating receptor. Thus, spontaneous domain separation appears to be a key feature of Gα, required but not sufficient for GDP release, yet terminated after GTP binding to initiate long-range conformational changes within Gα.^4,5^
2. GTP binding to the nucleotide binding pocket is communicated throughout the G protein by interconnected residue networks in a stepwise manner. The resultant unwinding of helix 5 and its reformation with a changed helical register disrupts the C-terminal Gα residues known to dock into the transmembrane core of active receptors. The main interface is substantially altered, destabilizing the interaction between G protein and receptor.
3. The disengagement of the G protein from the receptor is directional with a turning motion away from the receptor. Intriguingly, this newly delineated mechanism appears to be just the reverse to the large-scale conformational changes observed during molecular dynamics (MD) simulations for GPCR-stimulated GDP release.^5^ The authors suggest a corkscrew binding and unbinding pattern to underly G protein nucleotide exchange by GPCRs.
4. MD simulations using cryo-EM structures show that active-to-inactive state transition of the receptor seems to be mainly set in motion by the breaking of G protein-receptor contact, as both happen in concert. This transition is marked by the inward motion of TM6 and the movement of the bound ligand c-Epi towards the cytosolic entry site of the ligand binding pocket. It happens rather quickly and directionally, suggesting no inbuilt memory function for prior activation.

GDP-GTP exchange is foundational to GPCR-induced and G protein-mediated signal transduction. How GPCRs succeed in convincing their major transducer to progress from an inactive to an active, signaling-competent state is a choreography, investigators must have dreamed of visualizing in 3D since the first freeze-frame pictures provided poignant images into the signaling ballet.^2,3,5^ Although Skiniotis and his team were challenged with Gs proteins and the β2AR in an environment that is not entirely conducive to cinematography, they succeeded in ordering many poignant snapshots of Gs proteins after GTP binding to visualize the highly dynamic process of Gs protein activation by the β2AR. The 3D movie is not ready yet, however, its smell is already in the air.
